# Micro‐ and Nanopatterning of Highly Conductive PEDOT Thin Films

**DOI:** 10.1002/marc.70350

**Published:** 2026-07-02

**Authors:** Francesco Bisio, Sinan Baran Akdemir, Vittorio Bonino, Fabio Michieletti, Natascia De Leo, Valentina Brunella, Davide Comoretto, Angelo Angelini, Katia Sparnacci

**Affiliations:** ^1^ Dipartimento di Scienze e Innovazione Tecnologica (DISIT) Università degli Studi del Piemonte Orientale “Amedeo Avogadro”; INSTM UdR Alessandria via Michel 11 Alessandria 15122 AL Italy; ^2^ Advanced Materials Metrology and Life Sciences Division Istituto Nazionale di Ricerca Metrologica Turin Italy; ^3^ Dipartimento di Chimica Università degli Studi di Torino Torino Italy; ^4^ Dipartimento di Chimica e Chimica Industriale Università degli Studi di Genova Genova Italy

**Keywords:** optical lithography, oxidative chemical polymerization, micro‐ nanopatterning, nanosphere lithography, PEDOT, thin films

## Abstract

This work presents two fabrication strategies for the development of organic thin‐film devices at the micro‐ and nano‐scales. We make use of non‐commercial PEDOT synthesized via liquid‐phase oxidative chemical polymerization, using vanadium pentoxide (V_2_O_5_) as a oxidant. The optimization of this synthetic technique enables the production of homogeneous PEDOT films with a controlled thickness and tunable electrical properties. At the micro‐scale, we fabricate a PEDOT microstripe with gold contacts using standard optical lithography and confirm the high electrical performance of the processed PEDOT device. Moreover, at the nanoscale we apply a self‐assembling technique, namely the nanosphere lithography, to pattern the thin film over a large area with a hexagonally close‐packed array of nanopillars. This technique allows us to achieve individual nanopillar dimensions as small as 255 nm in diameter. The ability to achieve such controlled geometries suggests significant potential for developing organic photonic crystals and metasurfaces. These findings highlight a versatile fabrication framework that bridges standard lithography and unconventional self‐assembly, providing a scalable pathway toward advanced organic electronic and tunable photonic devices.

## Graphical abstract

This work presents micro‐ and nanoscale fabrication strategies for organic thin‐film devices using non‐commercial PEDOT synthesized by V_2_O_5_‐mediated oxidative polymerization. Optimized films show controlled thickness and tunable conductivity. At the nanoscale, nanosphere lithography enables large‐area hexagonally packed PEDOT nanopillar arrays down to 255 nm, highlighting potential for scalable organic photonic crystals and metasurfaces.

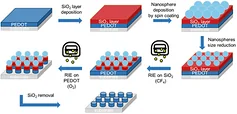

## 1 Introduction

Conductive polymers (CPs) have attracted sustained interest as key materials for organic electronic devices, offering lightweight, mechanically flexible, and low‐temperature‐processable alternatives to conventional inorganic semiconductors. Their tunable electrical properties and compatibility with large‐area fabrication techniques have enabled applications ranging from organic photovoltaics and light‐emitting diodes to energy storage and emerging nanostructured electronic devices [1, 2, 3].

Among CPs, poly(3,4‐ethylenedioxythiophene) (PEDOT) is one of the most widely used and extensively studied materials due to its high electrical conductivity in the oxidized state, optical transparency in the visible range, and excellent environmental stability [4]. These properties, combined with its chemical robustness, have established PEDOT as a benchmark material in organic and hybrid electronic devices [5, 6, 7]. However, a significant limitation of PEDOT lies in its poor processability. Being both insoluble and infusible, in fact, it is difficult to process into thin films. To address this, a water‐soluble polyanion, poly(styrenesulfonate) (PSS), is commonly employed as a doping agent and counterion during EDOT polymerization. This results in PEDOT:PSS, a commercially available and stable aqueous dispersion with outstanding processability and compatibility with a wide range of coating and printing techniques. However, the integration of PSS introduces distinct drawbacks. Excess PSS acts as an insulating barrier and induces morphological heterogeneity through phase separation between PEDOT‐rich and PSS‐rich domains, thereby limiting the material's intrinsic electrical conductivity. Consequently, PEDOT:PSS films typically require post‐deposition treatments for high‐performance applications. These treatments work primarily by removing some of the excess insulating PSS phase and inducing a conformational change in the PEDOT chains that facilitates charge transfer [8]. The most common treatments use polar organic solvents or strong acids. For example, the baseline conductivity of PEDOT:PSS (∼0.3 S cm^−^^1^) can be markedly enhanced to 1360 S cm^−^^1^ and via methanol treatment, and up to 2980 S cm^−^^1^ after superacid treatment [9, 10, 11]. Alternatively, PEDOT can be obtained directly as a thin film through the polymerization of 3,4‐ethylenedioxythiophene (EDOT) using several established approaches, among which oxidative chemical polymerization is the most widely employed [12]. This method can be implemented via either vapor‐phase or liquid‐phase routes. Vapor‐phase polymerization techniques enable the formation of highly ordered PEDOT films with superior electrical conductivity (up to 5000 S cm^−^^1^), owing to enhanced chain packing and structural order [13, 14].

However, these methods typically require complex equipment and stringent control over processing conditions, limiting their scalability and broader applicability [13]. In contrast, solution‐based oxidative polymerization offers greater simplicity and versatility, allowing PEDOT films to be deposited under mild conditions with precise control over thickness and morphology [15]. In this process, the monomer is mixed with or placed in contact with a solution containing an oxidant. The most commonly used oxidizing agents are iron(III) salts featuring small‐molecule counterions, such as iron chloride (FeCl_3_), iron p‐toluenesulfonate, (Fe(Tos)_3_), and iron trifluoromethane sulfonate (Fe(OTf)_3_). However, their tendency to crystallize often leads to structural defects that are detrimental to the resulting polymer's conduction properties. As an alternative, vanadium pentoxide (V_2_O_5_) is a potent oxidant that is highly active in initiating EDOT polymerisation. Its main reported applications concern the use of solid V_2_O_5_ as a reactive template in the preparation of high‐performance organic–inorganic hybrid nanocomposites, such as nanofibres [16] and nanowires and nanobelts [17]. It has also been used as an oxidant in the liquid phase to produce films with high conductivity (up to 1400 S cm^−^^1^) [15, 18].

Beyond bulk film properties, the functional performance of PEDOT is highly dependent on its nanoscale structure. Microstructural parameters such as chain packing, crystallinity, porosity, and surface roughness directly govern charge carrier transport, ion diffusion, and interfacial interactions [19]. Consequently, nanopatterning has emerged as a robust strategy to enhance charge transport, increase effective surface area, and improve interfacial properties [20]. Crucially, the execution of nanopatterning fundamentally diverges depending on whether the target is a soft, hydrated hydrogel matrix or a rigid, solid‐state thin film. For conductive PEDOT:PSS hydrogels—which are widely used in bioelectronics for their mixed ionic/electronic conductivity and biocompatibility— patterning is achieved not by material removal, but through *phase reorganization*. Laser‐induced phase separation offers a straightforward method for patterning these materials by locally rearranging the PEDOT‐ and PSS‐rich domains, converting irradiated regions into stable, conductive hydrogel tracks. This non‐destructive, localized approach enhances the wet conductivity, adhesion, and mechanical robustness of the hydrogel on polymer substrates, achieving wet conductivities of approximately 10^1^ S cm^−^^1^ with spatial resolution down to 5 µm [21, 22, 23].

In stark contrast, patterning solid‐state PEDOT thin films requires defining rigid geometric features through traditional additive or subtractive microfabrication. To fabricate PEDOT nanostructures, researchers have explored both bottom‐up approaches—such as template‐assisted growth and electrochemically induced porous morphologies—and top‐down techniques, such as optical lithography, electron‐beam lithography, and nanoimprint lithography [20, 24, 25]. Although bottom‐up methods facilitate the generation of high‐surface‐area architectures, they often provide limited control over long‐range order and pattern uniformity [11, 26]. In contrast, top‐down approaches, specifically lithography‐based techniques, offer superior control over feature size, periodicity, and spatial organization. Among these, nanosphere lithography (NSL) has gained significant attention as a scalable and cost‐effective method for producing highly ordered nanostructures over large areas [27, 28].

This technique exploits the self‐assembly of highly monodisperse colloidal nanospheres as an etching mask and combines it with reactive ion etching, enabling precise tuning of nanostructure geometry [14, 29]. Monolayers of nanospheres arranged in a hexagonal pattern over large surfaces can easily be obtained using several methods, including drop coating, dip coating, spin coating, and self‐assembly at the air–liquid interface [29].

However, the reliable formation of uniform nanosphere monolayers on conductive polymer surfaces remains challenging due to surface chemistry and wettability constraints.

In this context, this study explores the use of different lithographic techniques to fabricate PEDOT‐based devices on the micro and nano scales.

To this purpose, thin PEDOT films were prepared on a glass surface via a liquid‐phase oxidative polymerization process, based on the sequential deposition of a vanadium pentoxide (V_2_O_5_) oxidant solution and EDOT monomer onto glass substrates. This technique allows for the production of homogeneous PEDOT thin films with controlled thickness and tunable conductivity. These films were patterned using two different lithographic approaches. A conventional top‐down photolithographic approach, combined with metal deposition, was employed to fabricate a PEDOT microstripe with gold contacts. This micron‐sized device was then employed to characterize the electrical conductivity of the processed material.

Subsequently, a bottom‐up fabrication strategy, based on nanosphere lithography, was developed to fabricate well‐defined, vertically aligned PEDOT nanopillars, arranged in hexagonal bidimensional arrays over large areas [28, 29]. This combined approach provides insight into the relationship between solution‐based synthesis, film morphology, and nanoscale patterning.

This work highlights the potential of PEDOT as a versatile material for both electronic and photonic active devices.

## 2 Results and Discussion

PEDOT thin films were synthesized on glass substrates via a liquid‐phase oxidative chemical polymerization process [15], in accordance with a synthetic procedure previously reported [18], which is detailed in the Supporting Information and illustrated in Figure S1.

Briefly, the process involves the sequential deposition by spin coating of an oxidant solution and a monomer solution. The oxidant solution consists of vanadium pentoxide (V_2_O_5_) dissolved in methanesulfonic acid (MSA) at a concentration of 0.2 M, with the addition of a weak base (pyridine) to suppress undesired acid‐catalyzed side reactions. The monomer solution consisted of EDOT in acetonitrile at a concentration of 30% by volume. Upon addition of the monomer polymerization occurs almost instantaneously, yielding continuous and homogeneous PEDOT films. After synthesis, the films were rinsed in methanol and dried on a hotplate at 80°C.

Preliminary testing indicated that the concentration of the oxidizing solution and the deposition time, prior to monomer addition, are the two parameters most significantly influencing film characteristics. Specifically, increasing the concentration of the oxidizing solution at a constant deposition time resulted in thicker films. Conversely, at a fixed concentration, an increase in deposition time led to the formation of thinner films [18].

Consequently, the synthetic conditions were adjusted to obtain film thicknesses between 100 and 170 nm, as shown in Table S1. The electrical conductivity of the PEDOT films was determined using the four‐point probe (4PP) technique, and the obtained values are reported in Table S1. In the analyzed samples, electrical conductivity appears to be independent of film thickness and ranges from 154 to 202 S·cm^−^^1^. These results demonstrate that this liquid‐phase polymerization route enables precise thickness control and tunable electrical properties, providing a robust platform for subsequent nanopatterning.

### 2.1 Fabrication of PEDOT Microstripes

The micro‐scale patterning of PEDOT thin films was evaluated using a conventional top‐down photolithographic approach. The process is schematically described in figure 1a and detailed in the Methods section: the PEDOT thin film is spin coated with a positive‐tone photoresist and then baked to remove solvents and stabilize the structure; selective exposure to UV light via direct laser writing defines the desired pattern, and the subsequent resist development removes resist from the unexposed region. The uncovered PEDOT region is then etched, and the sample is washed in acetone to remove the residual resist, leaving an isolated PEDOT microstripe. A second lithographic step followed by gold deposition allows to define the electrical contacts for the electrical characterization.

**FIGURE 1:**
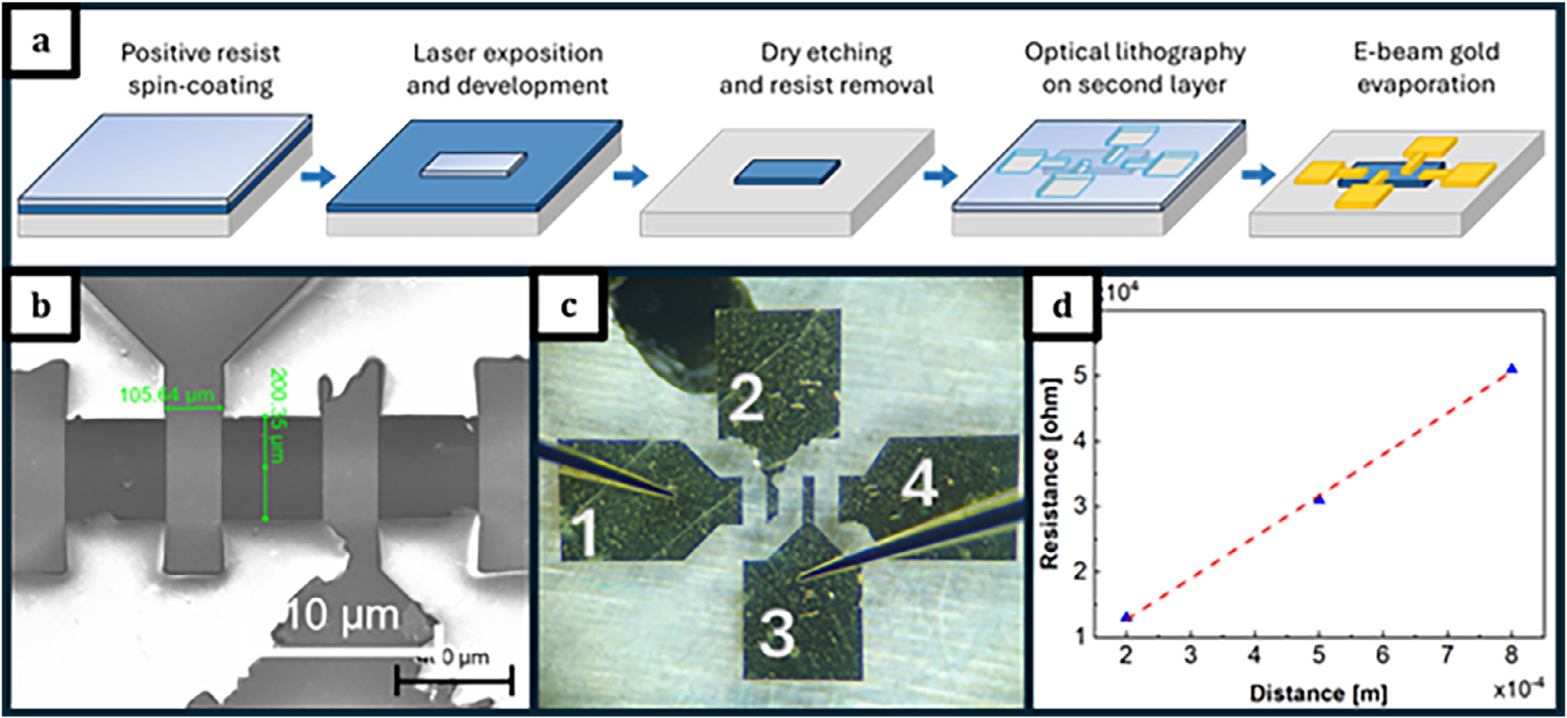
(a) PEDOT nanofabrication process scheme.(b) SEM micrography of EB‐deposited gold contacts on the PEDOT microstripe (c) Optical image of gold contacts and PEDOT microstripe under the probe station showing the electrical tips contacting electrodes 1–3. (d). Dependence of electrical resistance on the distance between electrical contacts.

This process is particularly challenging due to the exposure of the PEDOT to physical, chemical, and thermal stresses that can easily induce damage. We therefore characterized the electrical properties of the PEDOT microstripe in a probe station (details in the Methods section) and compared the results to the 4PP measurements on the PEDOT thin film. For this purpose, a PEDOT strip with a length of 1 mm, a width of 200 µm, and a thickness of 100 nm with four gold contacts was fabricated (SEM image in Figure 1b). The probe station allows a precise positioning of the electrical tips (Figure 1c) on the gold contacts (numbered from 1 to 4) to measure the I‐V curve and retrieve the PEDOT resistance. The results in figure 1d show the measured resistance when connecting contact 1 with the others, revealing a linear dependence of the resistance from the microstripe length.

The conductivity of the PEDOT strip was measured to be in the range of 11.5–11.6 S·cm^−^^1^, approximately one order of magnitude lower than that of the unpatterned bulk film (157 S·cm^−^^1^). Although we observe a loss of conductivity, the characterization confirms that the PEDOT is still showing good conductive properties after the fabrication process as compared to those commonly reported for functional conductive polymer nanostructures.

### 2.2 Fabrication of Ordered PEDOT Nanopatterns

A bottom‐up fabrication strategy, based on nanosphere lithography, was developed to fabricate well‐defined, vertically aligned PEDOT nanopillars, arranged in hexagonal bidimensional arrays over large areas.

The fabrication process is described in Figure 2, and at each stage the morphology of the sample surface is examined by scanning electron microscopy (SEM). As a first step we deposited a thin sacrificial layer of SiO_x_ over the PEDOT thin film. This step is essential for ensuring the surface uniformity, smoothness, and high wettability required to achieve a high‐quality, ordered nanosphere monolayer. Moreover, the SiO_x_ sacrificial layer ensures a good etching selectivity for the subsequent fabrication steps, as the RIE process to etch SiOx makes use of a specific gas (CHF_3_ + Ar) that does not degrade the polymers (see more details in the Methods Section). The sample surface was then uniformly covered with a monolayer of hexagonally close‐packed (HCP) polystyrene (PS) nanospheres (Figure 2a, see methods for details).

**FIGURE 2:**
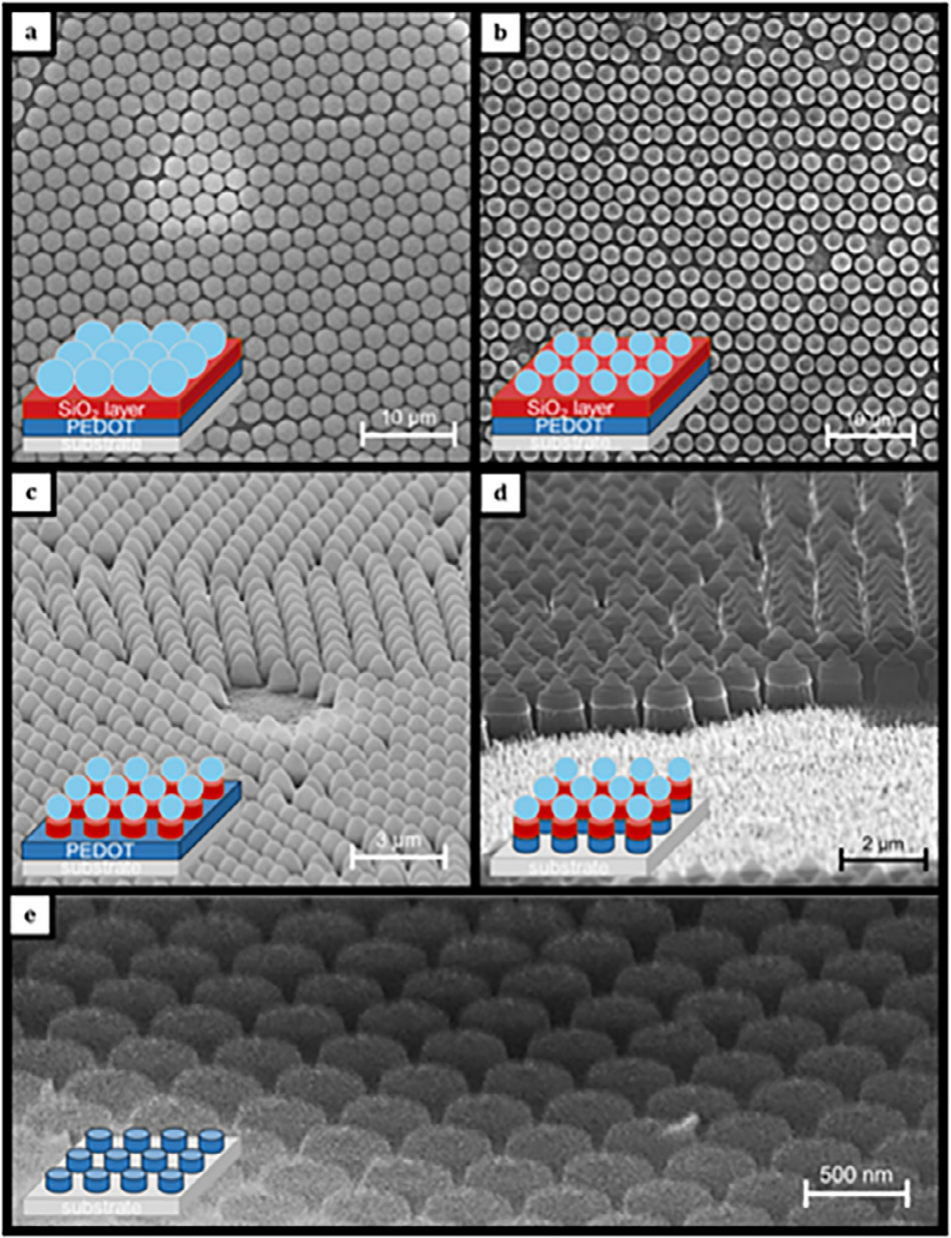
SEM micrographs of (a) PS nanospheres deposition, (b) nanospheres reduction, (c) silica layer etching, (d) PEDOT layer etching, and (e) PEDOT pillars.

Controlled plasma treatment was used to reduce the nanosphere diameter, thereby defining the lateral dimensions of the nanostructures (Figure 2b, Supporting Information S3.2).

The colloidal pattern was subsequently transferred into the SiO_x_/PEDOT stack by reactive ion etching in a two‐step process, yielding vertically aligned PEDOT nanopillars with diameters determined by the shrunk nanospheres and a periodicity inherited from the original colloidal lattice (Figure 2c,d). In the final step, the sacrificial SiO_x_ layer was removed by Ar/CHF_3_ plasma etching, leaving only the PEDOT nanopillars (Figure 2e, Supporting Information S3.3).

This approach enabled the reproducible fabrication of well‐defined, ordered PEDOT nanopillar arrays suitable for structural and functional characterization.

The morphology and in‐plane ordering of both the colloidal monolayer and the final PEDOT nanopillar array were analyzed from SEM images using a MATLAB‐based routine (Figure 3 and S3.4). The histograms reported in Figure 3c and S3.4c summarize this information by showing the angular distribution of the ordered domains: in these plots, the x‐axis corresponds to the orientation angle in the range 0–60°, while the y‐axis reports the percentage of domains in each angular bin. A narrow distribution centered around 0° therefore indicates that most domains are aligned along the reference direction, whereas broader histograms reflect a wider spread of orientations.

**FIGURE 3:**
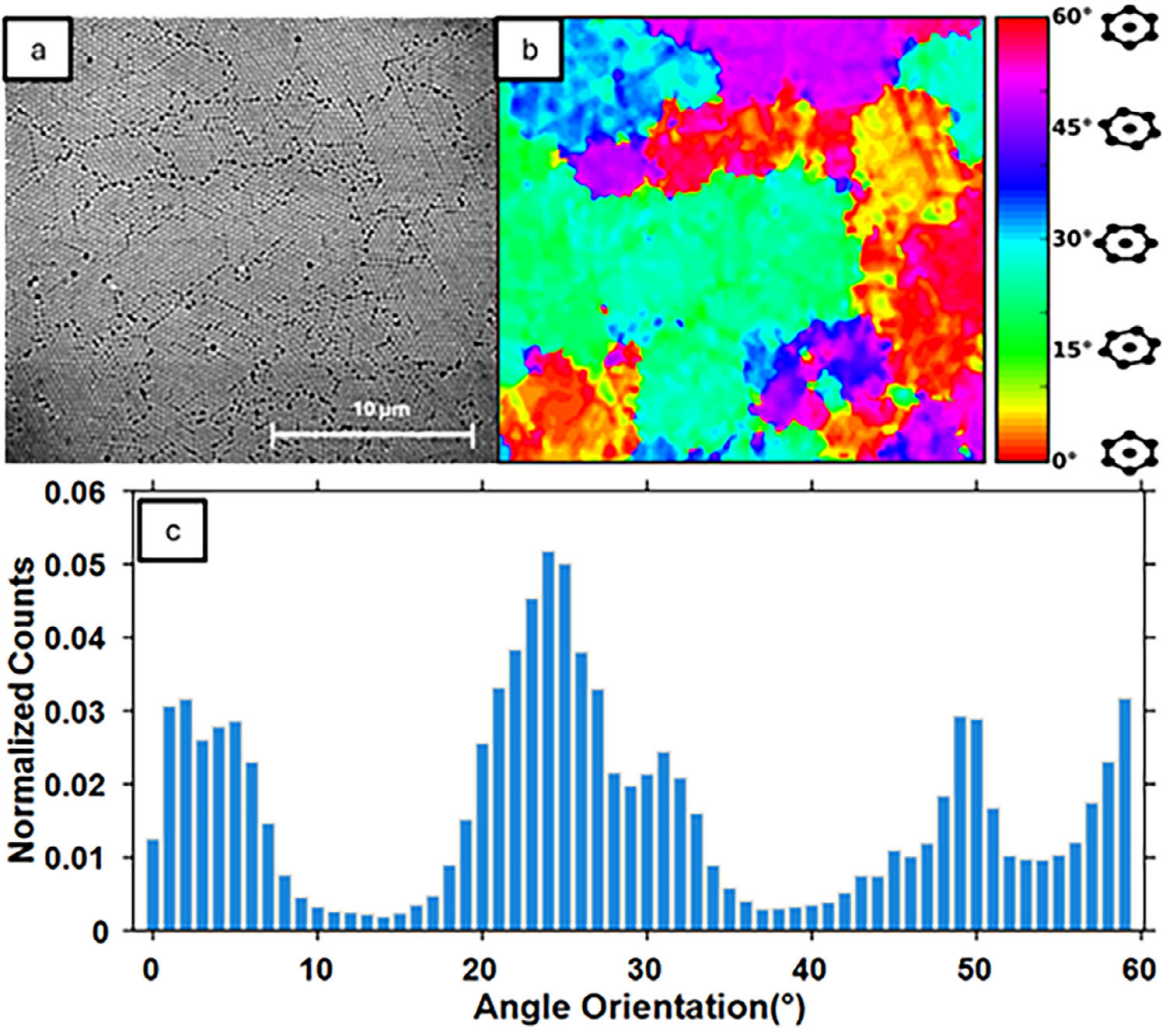
(a) SEM images After PEDOT etching (b) Colour map of the angular distribution: After PEDOT etching. (c)Histogram of the angular distribution of nanospheres ordered domains, x axis corresponds to angles from 0° to 60° and y axis corresponds to the normalized counts of domains: After PEDOT etching.

A MATLAB based routine (detailed in the Methods Section) analyzes the overall quality of the nanopillar array obtained. We analyzed both the initial nanosphere monolayer quality and the final nanopillar array to evaluate eventual loss of ordering due to the fabrication steps. For the colloidal mask, SEM images of the PS nanosphere monolayer on PEDOT/SiO_x_ (Figure S1.4a) show extended regions of close‐packed spheres with only a limited number of voids and multilayer defects. Quantitative analysis confirms this visual impression. Only 12.19% of the lattice sites deviate from sixfold coordination, and the two‐dimensional autocorrelation function yields a correlation length of 2980.87 nm [30]. After pattern transfer, SEM images of the PEDOT nanopillar arrays (Figure 3a) reveal that the global arrangement closely follows the initial colloidal lattice. In this case, the analysis (Figure 3b) shows a defect fraction of 13.14% and a correlation length of 2628.20 nm, with the grain orientation reported in Figure 3b,c. Thus, the long‐range hexagonal order is largely preserved, with only a modest increase in defect density and a slight reduction in correlation length. This demonstrates that the nanosphere lithographic process, combined with the thin SiO_x_ hard mask, effectively transfers the colloidal order into the PEDOT layer without substantial loss of structural quality. The comparison between the colloidal mask and the final nanopillar array highlights the key role of the SiO_x_ overlayer. Its surface quality and wettability properties enhance the overall quality of the nanospheres monolayer, while its higher hardness and etch resistance stabilize the pattern during the Ar/CHF_3_ and O_2_ plasma steps and help maintain the pillar shape. Moreover, the SiO_x_ caps left on top of the pillars provide additional mechanical protection to the organic core. Overall, the process yields PEDOT nanopillar metasurfaces with a controlled lateral size (≈255 nm), well‐defined pitch, and a high degree of hexagonal ordering, offering a robust platform for macromolecular and photonic application.

## 3 Conclusions

In this work we presented two different fabrication approaches to obtain PEDOT‐based devices on the micro and nano scales. The PEDOT thin films were obtained via a liquid‐phase oxidative chemical polymerization process using V_2_O_5_ as oxidant. This technique allows for the production of homogeneous PEDOT thin films with controlled thickness and tunable conductivity. By optical lithography combined with metal deposition, we fabricated a PEDOT microstripe with gold contacts to characterize the electrical conductivity of the processed material. Although we observed some loss in conductivity with respect to the pristine material, the characterization confirms that the microstripe preserves good electrical properties.

Furthermore, we show for the first time to the best of our knowledge that nanosphere lithography can be successfully applied to create an ordered monolayer mask with controlled geometry, and that the nanosphere pattern can be transferred to the organic thin film to obtain a well‐defined nanopillar HCP array over a large area, with individual elements diameter as small as 255 nm and an average grain size of 2.6 µm. We would like to emphasize that the experimental results demonstrate that the fabrication process does not result in a significant increase in defect density. This shows the potential for scaling up the process to larger areas, as well as the possibility of further improving the quality of the patterns by refining the initial nanosphere deposition step.

The experimental findings show that non‐commercial PEDOT thin films with properties that can be tuned at the polymerization stage can be structured at the micro and nanoscale using both standard lithographic techniques and unconventional self‐assembled techniques toward the fabrication of organic electrical and photonic devices.

In particular, the ability to achieve nanopillar arrays with controlled geometry paves the way for the development of organic photonic crystals and meta‐surfaces. The periodicity of the HCP structure and the sub‐micrometric dimensions of the elements suggest potential applications in visible light manipulation and organic plasmonic, where the optical response of the device can be tuned by modulating the oxidation state of the polymer.

## 4 Methods

### 4.1 Micropattering Process

The microfabrication of the samples used for conductivity measurements consisted of two optical lithography steps. In the first step, a positive‐tone photoresist (AZ5214e, Merk) was spin‐coated onto a 100 nm thick PEDOT film deposited on a SiO_2_ substrate. The soft‐bake temperature of the resist was adjusted accordingly to prevent thermal damage to the PEDOT layer. Exposure defined a window in the PEDOT, forming a single stripe of resist located at the center of the sample. The exposed PEDOT was removed by oxygen plasma etching, resulting in a single stripe electrically isolated from the surrounding film. In the second optical lithography step, four windows overlapping both the PEDOT stripe and the substrate were opened, followed by the deposition of a 200‐nm‐thick gold layer. A lift‐off process was then performed to remove the excess gold, yielding four gold contact pads electrically connected to the PEDOT stripe, as shown in Figure 1a.

### 4.2 Nanopattering Process

PEDOT thin films were first coated with a thin SiO_x_ hard mask in order to smooth the polymer surface and improve etch selectivity during subsequent processing steps. A SiO_x_ layer approximately 50 nm thick was deposited by electron‐beam evaporation under high vacuum and subsequently activated by oxygen plasma treatment to render the surface hydrophilic. A colloidal monolayer of polystyrene (PS) nanospheres (nominal diameter 300 nm, aqueous dispersion) was then assembled on top of the SiO_x_ layer using a two‐step spin‐coating protocol. This procedure yielded large‐area, hexagonally close‐packed (HCP) domains that served as a soft, bottom‐up lithographic mask.

The lateral dimensions of the nanostructures were controlled by isotropically shrinking the PS nanospheres in an O_2_/Ar plasma, reducing their diameter to approximately 255 ± 10 nm and opening well‐defined gaps between neighboring particles. The resulting colloidal pattern was transferred into the underlying SiO_x_/PEDOT stack by reactive ion etching (RIE) performed in two successive steps. First, an Ar/CHF_3_ plasma selectively etched the exposed regions of the SiO_x_ layer down to the PEDOT surface. Subsequently, an O_2_ plasma was used to etch the PEDOT through the SiO_x_ openings. Under these conditions, vertically aligned PEDOT nanopillars were obtained, with diameters defined by the shrunken nanospheres and a pitch inherited from the original colloidal lattice. The SiO_x_ caps were intentionally retained on top of the nanopillars to enhance their mechanical stability and protect the organic material during subsequent processing and characterization.

### 4.3 Characterization Methods

#### 4.3.1 Four‐Point Probe Technique

The electrical conductivity of the PEDOT films was determined using the four‐point probe (4PP) technique using an Ossila four‐point probe system with probe spacing 1.27 mm. System calibration is performed on the reference 100 nm ITO‐coated glass substrate (20×15 mm in size). The electrical characterization is carried out by means of a Keithley 6430 SourceMeter connected in a four‐terminal fashion to the four‐point probe setup. Electrical resistance is measured by performing an I‐V sweep, where current is forced between the extreme probing pins and the voltage drop is read across the inner pins. The injected current ramp extremes were set to ‐1 and 1 mA with a sweep rate of ∼1 mA s^−1^. Current source range was set to 1 mA and voltage reading range to 2 V.

#### 4.3.2 Two‐Terminal Electrical Characterization

The two‐terminal electrical resistance of PEDOT microstripes was measured in a EverBeing C‐4 probe station. Ever Being T20‐250 tungsten tips were contacted to gold pads through EverNeing‐005E micropositioners and connected to a Keithley 6430 SourceMeter in two‐terminal configuration. The electrical stimulation and measurement protocol described for the four‐point probe technique is applied for evaluating the resistance.

#### 4.3.3 Optical Profilometry

Film thickness was determined by step‐height analysis using a Sensofar S Neox optical profiler equipped with a confocal microscope. A scratch was introduced on the film surface using a fine needle to expose the underlying glass substrate. The height profile across this step is recorded to calculate the film thickness. As a typical example Figure S2 reports the optical profilometer image (a) and the step‐height profile (b) for sample S1C.

#### 4.3.4 Analysis of the Nanopillar Array Quality

In a first step, individual nanospheres (or nanopillars) are detected within a defined size window, and their centers are used to build a point lattice. Delaunay triangulation is then performed to identify local hexagonally close‐packed (HCP) domains and their grain boundaries. From this triangulation, a colour map is generated in which each colour corresponds to a different orientation of the local HCP unit cell (Figure 3b and S3.4b).

## Funding

PRIN2020 project Polymer mETamateriALs for nanophotonicS – PETALS – code 2020TS9LXS funded by the Italian Ministry of University and Research (MUR).

## Conflicts of Interest

The authors declare no conflicts of interest.

## Supporting information




**Supporting File**: marc70350‐sup‐0001‐SuppMat.docx.

## Data Availability

The data that support the findings of this study are available from the corresponding author upon reasonable request.
