# Supplementary material for: Micro‐ and Nanopatterning of Highly Conductive PEDOT Thin Films
Source: Macromol Rapid Commun. 2026 Jul 2;47(19):e70350. doi: 10.1002/marc.70350 (PMC13639208; doi:10.1002/marc.70350)
Supplement: Supplementary file 1 [file MARC-47-e70350-s001.docx]

Supporting Information

**Micro- and nanopatterning of highly conductive PEDOT thin films**

*Francesco Bisio, Sinan Baran Akdemir, Vittorio Bonino, Fabio Michieletti, Natascia De Leo, Valentina Brunella, Davide Comoretto, Angelo Angelini*, Katia Sparnacci**

### S1. Detailed synthesis of PEDOT thin films

PEDOT films were grown on glass substrates (15 × 15 mm) cut from Corning microscope slides. Prior to deposition, the substrates were treated with piranha solution (concentrated H₂SO₄ /30% H₂O₂ at a 3:1 volume ratio) at 80°C for 45 min to remove any contaminant and increase the hydrophilicity of glass surfaces. Subsequently, the substrates were rinsed thoroughly with Milli-Q water, dried in an N2 flow and used immediately.

The films were synthesized by a sequential solution polymerization method using vanadium pentoxide as the oxidant ^1^, as illustrated in Figure S1.

In details: the oxidant precursor solution was obtained by adding V_2_O_5_ (0.45g) in 12ml of methanesulfonic acid (MSA). The suspension was sealed in a vial, stirred 24h with a magnetic stirrer and then aged for 6 months at room temperature to ensure the complete solubilization of the vanadium pentoxide. The final oxidant solution was prepared by mixing 900 µL of the precursor solution with 420 µL of pyridine under stirring in an ice bath. The monomer solution was prepared by diluting EDOT in acetonitrile at 30% v/v.

The PEDOT films were then prepared as follows: the oxidant solution was spin-coated onto the treated substrates at 3000 rpm for 6 or 8 s (Table S1). Then, without interrupting the rotation, 200 μl of monomer solution was spin-cast for an additional 4 s.

**Figure S1**. Scheme of the synthesis process employed in the preparation of PEDOT films.

Upon addition of the monomer, the oxidant solution’s color rapidly shifted from yellow to dark blue, indicating the instantaneous polymerization of EDOT and the formation of the PEDOT film. Finally, the samples were washed with methanol to remove residual monomer and annealed at 80 °C for 10 min to enhance film stability.

**Table S1.** ((Thin film growth and electrical parameter values))

| Sample | Spin-coating velocity [rpm] | Oxidant solution concentration  [M] | Oxidant solution deposition time  [s] | Film thickness  by optical profilometer  [nm] | Conductivity by 4-point probe technique  [S/cm] |
| --- | --- | --- | --- | --- | --- |
| S1A (V1) | 3000 | 0,2 | 6 | 170 | 202 |
| S1B (V2) | 3000 | 0,2 | 6 | 150 | 185 |
| S1C (V3) | 3000 | 0,2 | 6 | 160 | 202 |
| S2A (V4) | 3000 | 0,2 | 8 | 110 | 186 |
| S2B (V5) | 3000 | 0,2 | 8 | 120 | 154 |
| S2C (P58) | 3000 | 0,2 | 8 | 100 | 157 |

### S2. PEDOT thin films characterization

Figure S2: a) Image of the step height analysis for sample S1C, b) step profile at x direction.

### S3. Detailed experimental parameters for PEDOT nanopillar fabrication

#### S3.1 SiOₓ hard mask deposition

After forming the PEDOT thin films on glass substrates ( Section 2), a SiOₓ layer was deposited by electron-beam evaporation under high vacuum to smooth the PEDOT surface and improve etch selectivity through a hard-mask strategy. A solid SiOₓ target was heated by a focused electron beam (30 mA), yielding a deposition rate of ≈0.4 Å s⁻¹ at a base pressure of ~7 × 10⁻⁷ mbar, as monitored by a quartz crystal microbalance. The SiOₓ thickness was set to ≈50 nm, which provided sufficient etch resistance while maintaining conformal coverage of the underlying PEDOT.

Prior to colloidal deposition, the SiOₓ surface was treated in an oxygen plasma (130 W, pure O₂) to render it hydrophilic and promote uniform wetting during spin coating of the polystyrene (PS) nanosphere dispersion.

#### S3.2 Colloidal mask assembly and nanosphere size reduction

Aqueous PS nanospheres with a nominal diameter of 300 nm were spin-coated onto the activated SiOₓ surface using a two-step spin program:

- Step 1: 500 rpm, acceleration 410 rpm s⁻¹, 20 s
- Step 2: 3500 rpm, acceleration 1150 rpm s⁻¹, 30 s

Under these conditions, extended monolayer regions were obtained, enabling the formation of large-area, hexagonally close-packed domains that act as a bottom-up lithographic mask.

Size reduction of the 300 nm spheres was performed in an Oxford PlasmaPro 100 Cobra plasma system. An O₂/Ar mixture (7.4 sccm O₂ / 24 sccm Ar, RF power 70 W, pressure 0.066 mTorr, 9 min) was used to induce rapid, essentially isotropic shrinkage of the PS particles. This treatment reduced the sphere diameter, efficiently broke the polymer “bridges” between neighboring spheres, and opened well-defined gaps. The final nanosphere diameter was ≈255 ± 10 nm, which defines the target lateral size of the PEDOT nanopillars.

#### S3.3 Pattern transfer by reactive ion etching

Pattern transfer into the SiOₓ/PEDOT stack was carried out in the same Oxford PlasmaPro 100 system by a two-step reactive ion etching (RIE) process.

Step 1 – SiOₓ etching
 The exposed SiOₓ regions between the shrunken PS nanospheres were etched using an Ar/CHF₃ plasma with the following parameters:

- RF power: 50 W
- Gas flows: Ar 33 sccm, CHF₃ 17 sccm
- Chuck temperature: 10 °C
- Pressure: 1.5 mTorr
- Etching time: 10 min

Owing to the higher hardness and etch resistance of SiOₓ, this etching time was required to fully open the mask down to the PEDOT layer while preserving the emerging nanopillar geometry. Residual PS material, when present on top of the SiOₓ pillars, effectively increased the overall mask thickness and contributed to its mechanical stability during this step.

Step 2 – PEDOT etching
 In the second step, the pattern was transferred into the PEDOT film using an O₂ plasma under conditions previously optimized for this material:

- RF power: 150 W
- ICP power: 30 W
- Gas flow: O₂ 25 sccm
- Chuck temperature: 10 °C

Under these conditions, the SiOₓ layer acted as a robust hard mask, enabling the formation of vertically aligned PEDOT nanopillars with diameters dictated by the shrunken nanospheres and a pitch inherited from the original colloidal lattice. The SiOₓ mask was intentionally left on top of the nanopillars after etching, as it improved structural stability and protected the organic material during subsequent handling and characterization.

Figure S3.3 PEDOT nanopillars obtained without the use of SiOx sacrificial layer. The nanosphere and the resist layer are still visible in the picture.

#### S3.4 MATLAB Analysis

Figure S3.4 a) SEM images After Nanosphere deposition b) Colour map of the angular distribution: After Nanosphere deposition c) Histogram of the angular distribution of nanospheres ordered domains, x axis corresponds to angles from 0° to 60° and y axis corresponds to the normalized counts of domains: After Nanosphere deposition

The colour scale from 0° to 60° represents the orientation angle of the domains with respect to a fixed in-plane reference direction; angles differing by multiples of 60° are crystallographically equivalent because of the sixfold symmetry of the lattice.

S4. Detailed experimental parameters for Fabrication of PEDOT microstripes

To pattern the PEDOT film into a single isolated microstripe, a positive-tone photoresist was applied and structured by direct laser writing.

1. O₂ plasma cleaning
    Samples were exposed to a short oxygen plasma cleaning step (40 W, 30 s, Plasma Matrix (Bdiscom) to remove adventitious contamination and promote uniform resist coating.
2. Photoresist spin coating (AZ5214E)
    A positive-tone resist AZ5214E was spin-coated for 30 s at 3000 rpm, yielding a resist thickness of approximately 1.5 µm .
    The coated substrates were then soft-baked on a hotplate at 110 °C for 3 min to remove solvent.
3. Exposure (direct laser writing)
    The stripe pattern was defined by direct laser writing using the Heidelberg μPG 101. The representative exposure condition was 10 mW, 100% intensity, no filter.
4. Development
    The exposed resist was developed using AZ developer diluted with water (AZ DEV: H₂O = 1:1) for 90 s, followed by rinsing with DI water and drying under N₂.

S4.1 PEDOT etching

After development, the patterned resist served as an etch mask and the exposed PEDOT regions were removed by oxygen plasma etching to isolate the microstripe with Oxford PlasmaPro 100.

RIE conditions:

- RF power: 150 W
- ICP power: 30 W
- Gas flow: O₂ 25 sccm
- Chuck temperature: 10 °C

S4.2 Photoresist stripping (after PEDOT etch)

Residual resist was removed by solvent stripping:

- Acetone soak: 65 °C, 15 min,

S4.3 Lithography step 2: fabrication of Au contact pads (lift-off)

Gold pads were defined in a second lithographic step to provide electrical contacts overlapping both the PEDOT stripe and the surrounding substrate.

1. Photoresist coating
    AZ5214E was spin-coated again (30 s at 3000 rpm) and soft-baked (110 °C for 3 min).
2. Exposure
    The pad pattern was written using Heidelberg μPG 101 with direct laser writing (representative: 10 mW, 100%, no filter).
3. Development
    Development was performed using AZ DEV : H₂O = 1:1 for 90 s followed by rinse and N₂ drying.
4. Gold deposition
    A gold layer of 200 nm thickness was deposited by vacuum evaporation (e-beam evaporation).
5. Lift-off
    Lift-off was performed by soaking the sample in acetone at room temperature for ~24 h. Ultrasonication was avoided to prevent damage to the PEDOT stripe and to reduce the risk of pad/stripe delamination.

(1)	Chen, R.; Sun, K.; Zhang, Q.; Zhou, Y.; Li, M.; Sun, Y.; Wu, Z.; Wu, Y.; Li, X.; Xi, J.; Ma, C.; Zhang, Y.; Ouyang, J. Sequential Solution Polymerization of Poly(3,4-Ethylenedioxythiophene) Using V2O5 as Oxidant for Flexible Touch Sensors. iScience 2019, 12, 66–75. https://doi.org/10.1016/j.isci.2019.01.003.
